# Text Messaging to Enhance Behavioral Health Treatment Engagement Among Justice-Involved Youth: Qualitative and User Testing Study

**DOI:** 10.2196/10904

**Published:** 2019-04-05

**Authors:** Marina Tolou-Shams, Juliet Yonek, Katharine Galbraith, Eraka Bath

**Affiliations:** 1 Division of Infant, Child and Adolescent Psychiatry Zuckerberg San Francisco General Hospital University of California, San Francisco San Francisco, CA United States; 2 Department of Psychiatry Weill Institute of Neurosciences University of California, San Francisco San Francisco, CA United States; 3 Department of Psychiatry and Biobehavioral Sciences Jane and Terry Semel Institute for Neuroscience and Human Behavior University of California, Los Angeles Los Angeles, CA United States

**Keywords:** juvenile delinquency, treatment adherence and compliance, mental health, short message service text messaging

## Abstract

**Background:**

Mental health and substance use disorders are highly prevalent in justice-involved youth, yet only 8% of court-involved, nonincarcerated (CINI) youth in need of treatment receive it. Dual diagnosis (co-occurring psychiatric and substance use disorders) in justice-involved youth is highly predictive of recidivism. Identifying novel approaches, such as the use of mobile health (mHealth) technologies, to close this gap between need and receipt of behavioral health treatment for the CINI population could potentially offset rates of reoffending into adulthood. Text-messaging (short message service, SMS) interventions have demonstrated efficacy in improving treatment adherence and other associated outcomes in other vulnerable youth populations, but development and testing of mHealth interventions to improve behavioral health treatment rates and outcomes for CINI youth are lacking.

**Objective:**

This study aimed to collect qualitative data from key stakeholders to inform the development of a theoretically grounded, family-based text-messaging (SMS) intervention targeting CINI youth’s behavioral health treatment engagement; additionally, the aim was to conduct end-user testing over 6 months with CINI youth and caregivers to determine intervention feasibility and acceptability.

**Methods:**

CINI youth and caregivers were referred from a California-based Juvenile Probation Department and community-based provider organizations providing services for justice-involved youth. Eligibility criteria included the following: being a justice-involved youth or a caregiver of a justice-involved youth, English speaking, youth aged 13 to 17 years old and either referred to or currently attending mental health or substance use treatment, and youth and caregiver have access to a cell phone with text-messaging capability.

**Results:**

Overall, 28 individuals participated in focus groups and interviews—8 youth, 5 caregivers, and 15 juvenile justice (JJ) personnel. Three major themes emerged: (1) texting among JJ personnel and CINI youth and caregivers in their caseload is common but not systematic, (2) stigma and privacy are perceived as barriers to texting youth about behavioral health treatment appointments, and (3) messages should be short, simple, relatable, positive, and personalized. In total, 9 participants (7 youth and 2 caregivers) participated in end-user testing and rated the intervention as useful, helpful, and supportive.

**Conclusions:**

Text messaging (SMS) is an acceptable and feasible means of reminding CINI youth to attend behavioral health treatment appointments. Future implementation challenges include making text messaging (SMS) personalized and tailored but not resource intensive (eg, requiring one-to-one, 24/7 human contact) and identifying which systems will deliver and sustain the intervention. Text messaging (SMS) among justice personnel, youth, and their caregivers is already widespread, but lack of clear guidelines about privacy, confidentiality, and information sharing poses ethical conundrums. Future hybrid-type research designs that explore the efficacy of the intervention while also studying ethical, system, and policy-level factors associated with using digital health interventions to improve CINI youth outcomes is a key next step.

## Introduction

### Background

Mental health and substance use disorders are significantly more prevalent among justice-involved youth than their nonoffending counterparts [1-3]. Among detained youth, it is estimated that approximately 70% to 90% of these individuals have psychiatric symptoms [2,4,5]. Court-involved, nonincarcerated (CINI) youth—those who are legally involved but living in the community—are also more likely to have psychiatric problems compared with general community adolescent samples; between one-third and one-half of this population has a diagnosable psychiatric condition [6]. In addition, 73% have some form of lifetime trauma exposure [7], and approximately 50% of first-time offending youth endorse lifetime marijuana use [8]. Despite high rates of behavioral health (mental health and substance use) disorders among justice-involved adolescents, only 15% of detained youth receive behavioral health treatment; this number falls to 8% once these youth reenter the community [2]. These statistics regarding treatment receipt among justice-involved youth are important to consider from a health care perspective and in terms of public health significance and policy. Dual diagnosis (ie, cooccurring psychiatric and substance use disorders) in justice-involved youth is one of the most significant predictors of recidivism [9], and as such, closing the gap between need and receipt of behavioral health treatment for justice-involved youth could potentially offset rates of reoffending into adulthood [10].

Several barriers contribute to the gap between need and receipt of behavioral health treatment. CINI youth are commonly diverted at various points within the system (eg, arrest, intake, and probation) and referred to different providers and systems throughout their community while still under court supervision. Thus, screening and further assessment of their initial behavioral health needs are not implemented as easily, systematically, or consistently as might be implemented in juvenile detention settings, where all youth come through a central intake. Furthermore, formal assessment does not guarantee referral to treatment, and unfortunately, many of those referred to treatment do not link to providers to initiate treatment (ie, attend the first appointment), particularly when referrals require complex navigation through the community. Even among those who do successfully initiate mental health treatment, only some remain engaged in treatment (defined as at least two visits within 60 days of the first appointment) and continue care (defined as minimum of 3 months of treatment) [11]. From initial intake into the system through the continuum of care, there are a host of barriers that contribute to low rates of treatment receipt among justice-involved youth; these include youths’ developmentally appropriate lack of insight, lesser problem recognition and motivation to engage in treatment [12-14], gaps in communication between the justice system and the community-based organizations that provide mental health services, overburdened systems with excessive waiting periods of appointment times, and staff turnover and burnout [11,15]. Racial and ethnic minority youth with fewer individual, family and neighborhood resources are also disproportionately represented in the juvenile justice (JJ) system, thereby presenting additional barriers to behavioral health services access and engagement [16,17]. Justice-involved youth and caregivers also have the additional context of justice system oversight and involvement (eg, mandated treatment, punitive sanctions-based approach to noncompliance) that may or may not affect treatment engagement as compared with nonjustice-involved populations. A review of studies among adult justice-involved populations suggests that mandated or sanction-based approaches overall are not effective in improving substance use or criminal justice outcomes [18]; thus, the field should identify ways to reduce reliance on compulsory approaches to enhancing treatment engagement and outcomes.

Mental health treatment engagement is predictive of improved behavioral health outcomes [19], and thus working to improve rates of treatment receipt by enhancing treatment engagement is critical. The use of digital mobile health (mHealth) technologies has been shown to be a low-cost, efficacious way of reaching vulnerable populations to facilitate treatment engagement [20]. For example, a recent meta-analysis (N=14 studies) concluded that short message service (SMS) text messaging is a promising tool for effective substance use prevention (including relapse) for nonoffending adolescents and young adults [21]. In another study, the use of bidirectional SMS text messaging with caregivers to enhance adolescents’ receipt of vaccine and well-care services improved adolescents’ utilization of both services [22], suggesting that caregiver involvement in adolescent-focused mHealth interventions may also be effective in improving other outcomes (eg, engagement in mental health treatment). Studies show that only some, but not all, caregivers are ready for electronic messaging support for health care [23] and that depending on caregiver race (eg, Latino), socioeconomic status (eg, low), and age (eg, younger), SMS text messaging may be more or less appealing as a tool for their adolescent’s health care engagement [19,24].

The use of mHealth technology presents a promising approach for closing the gap between CINI youth’s need and receipt of behavioral health treatment. Mobile phone usage among justice-involved youth, particularly those supervised in the community, is also widespread [25]. However, to our knowledge, an empirically supported mHealth technology intervention specifically tailored to justice-involved youth and their caregivers does not currently exist.

### Objectives

This study developed and conducted end-user testing of a dyadic (youth and caregiver) SMS text-messaging intervention that included sending appointment reminders and motivational messages to enhance likelihood of the youth attending face-to-face community-based treatment, as referred by probation staff. Our aim was to understand what key system stakeholders (clinicians and probation staff) and end users (youth and their families) thought was feasible and acceptable regarding the use of SMS text messaging to enhance treatment engagement.

## Methods

### Study Overview, Population, and Recruitment

Pilot study data collection was completed in 2 phases: (1) Development (focus groups and interviews with CINI youth, caregivers, and JJ personnel to inform the SMS text-messaging intervention) and (2) End-User Testing (with CINI youth and caregivers). For both phases, youth and caregivers were referred from a California-based Juvenile Probation Department and community-based provider organizations that served justice-involved youth and their caregivers. Probation staff and community-based providers referred interested youth and families to the study staff, and the study staff screened referred youth and caregivers for eligibility. Youth eligibility criteria included being English speaking, between 13 and 17 years old, justice involved, either referred to or currently attending mental health or substance use counseling, and have a personal cell phone with SMS text-messaging capability. Caregiver eligibility included being English speaking and have a personal cell phone with SMS text-messaging capability. JJ personnel included any probation staff and providers (eg, case managers, behavioral health clinicians, and social workers) serving justice-involved youth and their families in the same geographical region as youth and caregivers in the study. JJ personnel were recruited through emails and follow-up phone calls, with assistance from JJ administrators. In the Development phase, caregivers provided written informed consent for their or their youth’s participation and youth provided separate assent. JJ personnel provided written informed consent for their participation. In the End-User Testing phase, because of challenges with reaching caregivers in person, parental consent was waived for youth participation and youth completed in-person written consent. For youth who had involved caregivers and gave permission to the study staff to contact them, interested caregivers provided verbal consent (by phone) for their separate End-User Testing phase participation. Institutional Review Board approval for the study was obtained from the Principal Investigator’s (PI’s) institution before any data collection.

### Study Procedures: Development Phase

In total, 4 focus groups (1 JJ probation staff, 1 JJ providers, and 2 youth) and 5 individual caregiver phone interviews were conducted between October 2016 and February 2018. Before starting focus groups and interviews, participants completed a brief demographics questionnaire. The focus group and interview guides were developed by the study PI (MTS), a child psychologist with expertise in designing and implementing behavioral health interventions targeting justice-involved youth as well as qualitative methodology, and a health services researcher with expertise in qualitative methodology (JY). Focus groups and interviews were conducted by the PI and 2 research staff members, all of whom were trained in both qualitative data collection methods. Focus groups were either conducted in a private conference room in the probation department (probation staff) or community partner settings (providers and youth). All interviews and focus group sessions were audio recorded with consent. The youth and JJ staff focus groups each lasted for approximately 90 min, and the individual caregiver phone interviews lasted for 60 min. Youth and caregivers were each compensated US $25 for the focus group and individual interviews, respectively. JJ personnel were compensated US $25 for their participation, if allowable by their organization.

### Focus Groups: Youth

Groups began with an *ice breaker* section regarding participants’ general cell phone use and texting patterns with JJ personnel and caregivers. Next, youth were asked about the types of messages they would find most helpful or effective in increasing their attendance and engagement in mental health and substance use treatment. For example, participants were asked about messages serving as appointment reminders, messages about the benefits of completing treatment and other probation-related requirements on time, and messages providing positive reinforcement for attending appointments. Facilitators also asked participants for specific feedback regarding SMS text message structure, including message frequency, language, level of interactivity, and possible concerns, such as privacy and participant burden.

### Focus Groups: Juvenile Justice Personnel

Groups began by asking personnel to provide their perspective on the acceptability, practicality, and feasibility of administering an SMS text message–based system within a probation department or a treatment setting. Participants were asked about their current texting practice with youth or caregivers as well as to describe the individual, family, and system-level benefits to and challenges associated with using an SMS text-messaging system to send systematic appointment reminders and motivational messages to youth.

### Interviews: Caregivers

Caregivers were first asked to describe their level of involvement in their youth’s court and treatment-related appointments, including their communication with youth about appointments. They were also asked to describe their preferred mode of communication with their child (eg, phone, email, and text) as well as perceived barriers and benefits to using SMS text messaging to remind youth of their appointments and keep them engaged in treatment. Caregivers were also asked to describe their interaction and communication with their youth’s probation officers or clinical providers, including preferred forms of communication (texting, phone, and in-person visits).

### Study Procedure: End-User Testing Phase

SMS text message development and content were informed by results from the Development phase. Participants directly received SMS text messages for a period of 6 months. Automated appointment reminders were sent to both CINI youth and caregivers 3 days before, 1 day before, and on the day of the appointment. A follow-up message was sent after the appointment to find out whether the youth attended their appointment, and if not, why not. Prescripted motivational messages were sent twice a week (eg, on Monday and Friday), and they did not include any words related to mental health or substance use (per phase 1 participant feedback).

Perceived usefulness, acceptability, and recommendations for improvement were assessed via repeat Web-based surveys over 6 months (administered at 1, 3, and 5 months). CINI youth and caregivers were asked for their general opinions about the intervention (ie, clear, helpful, and user-friendly), the motivational messages (ie, interesting, motivating, and boring), and the reminders (ie, helpful). Youth and caregivers received US $25 for each survey completed.

### Qualitative Data Analysis

Interviews, focus group recordings, and written notes were reviewed by research team members for accuracy and completeness. This information was used to construct an executive summary of the main discussion points and topics within 24 hours of conducting focus groups or individual interviews, and the information was used to identify commonly reported themes. Themes were refined on the basis of group discussions by the research team, led by the PI (MTS). Illustrative quotes were then extracted for each theme.

### Survey Data Analysis

Given the pilot nature of this intervention, participants completed surveys at 1, 3, and 5 months (within the 6-month intervention period) to inform iterative refinement and obtain feedback on changes over time. Descriptive statistics were used to summarize 5-month (final) follow-up survey results for CINI youth and caregivers who participated in user testing.

## Results

### Development Phase

There was a total of 28 study participants—8 youth, 5 caregivers, and 15 JJ personnel (8 probation staff and 7 providers). There was a single youth and caregiver dyad; the remaining youth and caregiver participants were not related. Sample youth and caregiver demographic characteristics are in Table 1.

### Key Development Phase Themes

**Figure 1:**
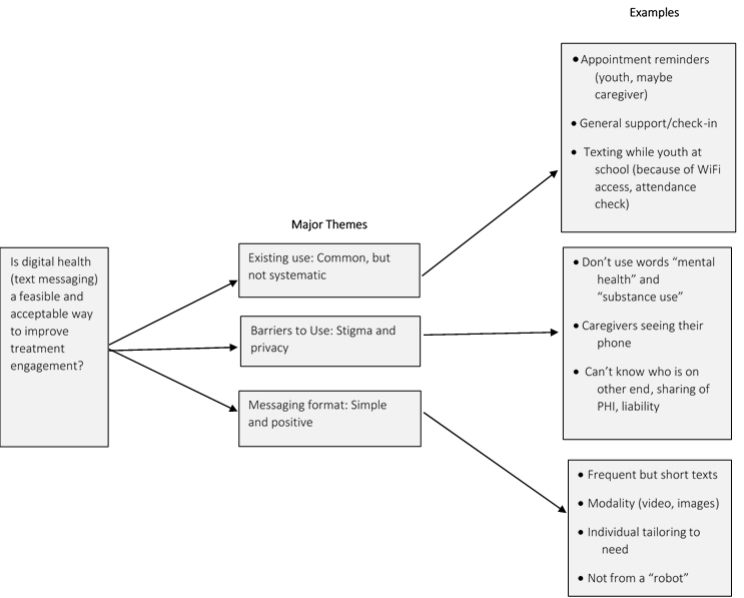
Primary themes on the feasibility of short message service text messaging for justice-involved youth. PHI: Personal Health Information.

In total, 3 major themes (Figure 1) emerged from the analysis of focus groups and interviews: (1) texting among JJ personnel and youth and caregivers in their caseload is common but not systematic, (2) stigma and privacy are perceived as barriers to texting youth about mental health and substance use treatment appointments, and (3) messages should be short, simple, relatable, positive, and personalized.

#### Theme 1: Texting Among Juvenile Justice Personnel, Youth, and Caregivers Is Common but Not Systematic

Youth, caregivers, and JJ personnel were universally enthusiastic about an SMS text message–based system to help remind and encourage youth to attend mental health or substance use treatment appointments and complete treatment. All youth said that they already use SMS text messaging to communicate with their JJ personnel contact (eg, probation officer, clinician, or case manager) primarily to check in, such as “They check up on me,” and “They ask how I’m doing in school,” but also to schedule appointments and to obtain general advice and support, such as:

One time I was at work and got really mad at a co-worker. I didn’t want to get in trouble so I texted my case manager for help. I got a text back helping me…telling me what to do.

I’ll text my case manager on a daily basis, if I have a question on something.

All caregivers (n=5) were highly interested and invested on obtaining SMS text message reminders of their youth’s responsibilities and mandates to support or monitor; however, 3 caregivers noted that they were not as facile as the youth in texting and that their youth often ignore their messages, which then results in a phone call (that some caregivers preferred over texting to begin with). One caregiver noted:

I’m not really a texter.

I’d rather talk [to my daughter] and get an answer right there instead of having to wait for the answer [via text].

JJ personnel said they commonly text youth to let them know they are trying to get a hold of them, to *check in* and to remind them of their appointments. However, the frequency of texting for these reasons varies for each child and thus is not systematic. JJ personnel commented, for example:

Some kids respond best to [a text message that says], “every Tuesday at 2 pm I will be here at your school.” But for some kids that’s not going to work. Everything needs to be very individualized for each kid. Their situations are so up and down that it could be one way for 2 months and then change in a completely different way.

They may meet Tuesday this week and Thursday next week. There’s a lot of fluidity in the scheduling because they’re all over the place.

In particular, the probation staff stated that they frequently use texting to communicate with caregivers about their teen’s appointments as they are more likely to respond to a text than a phone call. One probation officer explained:

I can depend on a text message to the parent to remind the kid more than a voicemail because I don’t know if they’re going to listen to it.

**Table 1:** Demographic characteristics of court-involved, nonincarcerated youth and caregivers.

Demographics		Development phase	End-user testing
Youth (n)		8	7
Median age (years)		17	16
**Gender,** **n (%)**			
	Female	6 (75)	1 (14)
	Male	2 (25)	8 (8)
**Race,** **n (%)**			
	White	0 (0)	0 (0)
	African American/black	3 (38)	2 (29)
	Asian	1 (13)	1 (14)
	Multiracial	2 (25)	0 (0)
	Native Hawaiian or other Pacific Islander	1 (13)	0 (0)
	American Indian or Alaska native	0 (0)	1 (14)
	Other	1 (13)	3 (43)
Hispanic ethnicity, n (%)		3 (38)	6 (86)
Owned a cell phone (yes), n (%)		6 (75)^a^	7 (100)
Caregiver (n)		5	2
**Relationship to youth**			
	Biological caregiver	4 (80)	2 (100)
**Age range,** **n (%)**			
	35-44	1 (20)	N/A^b^
	45-54	2 (40)	1 (50)
	55-64	1 (20)	1 (50)
	65+	1 (20)	N/A
**Gender,** **n (%)**			
	Female	4 (80)	2 (100)
**Race,** **n (%)**			
	White	0 (0)	1 (50)
	African American/black	4 (80)	1 (50)
	Asian	0 (0)	0 (0)
	Multiracial	0 (0)	0 (0)
	Native Hawaiian/other Pacific Islander	1 (20)	0 (0)
	American Indian or Alaska native	0 (0)	0 (0)
	Other	0 (0)	0 (0)
Hispanic ethnicity, n (%)		0 (0)	0 (0)
Owned a cell phone (yes), n (%)		5 (100)	2 (100)

^a^Owning a personal cell phone was only required for teens participating in the End-User Testing phase.

^b^N/A: not applicable.

JJ providers specifically expressed that automating text-based appointment reminders would be very helpful, particularly if reminders could be generated directly from the calendar on their cell phone or computer. Examples of suggestions made included:

With the Google calendar you can do a reminder and that’s very helpful. But if there was also a way to set that calendar up to text or send a text to whomever – as you put it in your calendar there’s an option to send a text – that would be very useful.

What would be helpful is being able to input all of my appointments scheduled for that week. Being able send those reminders weekly, when I’m setting it up in a calendar and then those messages with reminders are getting sent out so I don’t even have to think about it.

Entering appointment dates and times into a separate system felt like *additional work* and less efficient than sending appointment reminders themselves, especially given the frequency of 1-time (nonreoccurring) appointments. One JJ provider stated:

Going into a system that has to also be altered constantly…that feels like more work than sending out the text [directly]. I would have to figure out a system, what’s the schedule, what do I need to remind them of? It feels like almost an additional step. The large majority of the work we do is not “at every Tuesday at 2 PM we do this.”

#### Theme 2: Barriers to Use—Stigma and Privacy

Youth, caregivers, and JJ personnel were adamant that neither appointment reminders nor motivational messages should mention mental health or substance use. For example, one youth stated:

You have an appointment at such and such, but don’t say what it’s for.

A caregiver separately commented:

Instead say “hey, you have a session” or “hey, you have an appointment.”

JJ providers explicitly confirmed that sending youth a text directly asking about substance use or mental health would not be well received, as illustrated by statements such as:

That wouldn’t be really comfortable…because those are sensitive subjects and that sensitivity isn’t going to come across well through text. It is going to be really dry.

I haven’t thought of a time where I would have done that. I can’t even imagine texting like ‘are you okay?’ It would be more like, if I am concerned, I am calling. I am not going to text.

Although youth said they use the password feature to lock their phone, some reported sharing their password with certain friends and dating partners. Most also said that their caregivers tried to look at their phones at least once, even when they were not given permission to do so. One youth described when her parent tried to take her phone while sleeping:

One time my mom came in there when I was sleeping and was going through my phone, SnapChat, Twitter, she was just being nosy. I was so mad.

Another youth described why privacy was important (ie, that their phone held all of their sensitive information):

You’re not getting my phone. I have too much information up in here.

JJ personnel also expressed several concerns about youth privacy when sending texts, including that there is no way to know whether the phone is in the youth’s possession or who is on the other side of the text and whether it is the youth whom they are intending to contact:

Texts are there forever. If youth doesn’t have a passcode, then anyone can see it.

Concerns were also raised by probation staff, specifically that text conversations can also be subpoenaed, and there is currently no system-level policy or protocol on how to manage this. One probation officer stated:

Some of these kids have warrant searches on their phones…So a lot of times they don’t want to communicate over text.

Probation officers expressed concern over what aspects of SMS text messages are part of the legal record and what constitutes protected health information when shared over texting. JJ providers separately expressed similar concern over how to handle potentially self-incriminating information sent to them by the youth or caregiver. JJ providers additionally raised uncertainty and questions regarding whether text conversations with youth should be documented in the treatment chart as part of clinical care; this might be akin to written collateral contact logs often included in client charts as part of standard clinical practice to document any contact outside of scheduled appointments. For example, 1 provider asked the question:

Since the communication [via texting] is happening during work hours, what responsibility do we have to maintain those records of that communication?

Another JJ provider expressed concerns that *not* documenting text conversations might diminish perceptions of their actual workload:

Are we really keeping track of how much outreach and how much that we’re doing [via texting]? I can say no, we are not writing our [text conversations] in the file every time, and so then it doesn’t really look like we’re doing all of the stuff that we’re doing…yeah it might be on your phone, but that doesn’t do us any good when we’re talking about the work that we’ve done.

Finally, JJ personnel shared concern about how their own privacy can be compromised when using their personal cell phones to text youth, which all JJ personnel reported occurs by virtue of necessity and lack of system resources to provide work cell phones for probation or provider personnel.

#### Theme 3: Messages Should Be Short, Simple, Relatable, Positive, and Personalized

Youth and JJ personnel emphasized that messages should be short and conceptually straightforward. One youth raised the option of visual, Multimedia Messaging Service (MMS), or video messaging indicating that:

A video would be cool. Not longer than a minute though.

Multiple youth mentioned that SMS text language should be conversational and friendly, noninvasive, and worded in a way that sounds like something a teenager would say (eg, language that they can relate to), such as:

It has to be short and sweet

How they say it is important. Don’t just pile it on like you got this this this and this. Let me know what I have to do.

Another youth stated:

It would be cool if it’s an app that reminds you of your appointments. Not something that asks you questions all about your life.

Positively worded messages were universally endorsed by youth and caregivers. Youth stated that they would prefer texts emphasizing that it is their choice to attend mental health or substance use treatment appointments rather than being told what to do, such as:

It’s your choice. Get what you have to do to get things done so you can do what you want to do.

Caregivers provided examples of SMS text messages for appointments that they perceived might be motivating for their youth, including messages that reminded their youth of longer-term goals (eg, getting back to *normal life*, not having to worry as much), such as:

Get it over with so you don’t have to keep worrying, instead of making it last another 3 months.

The sooner we can get this behind us the sooner we can get back to our normal life.

Other suggestions made by youth and JJ probation staff specifically included having positive, nonjudgmental words in the texts, such as “Keep up the positive work. You’re really handling things well,” and 1 probation staff suggested, “Remind them of the positive… feeding to their strengths.” Similarly, JJ providers stressed the importance of *tone* to get kids’ attention and keep them engaged. Some staff use emojis (ie, ideograms and smileys used in electronic messages and Web pages) to convey a positive tone or use a humorous character to relay the message (eg, Mickey Mouse). For example:

The winky eye is probably the one I use the most, and then the one with the big old giant smile.

I use the hand waving one. It’s like giving them praise.

Youth universally stated that they wanted messages to be individualized and bidirectional rather than generic, even if automated. They expressed a desire for messages to come from an actual person, not a *robot*:

I would rather have somebody actually texting me on the other side and they would reply when they got my texts.

Caregivers (n=4) and JJ providers expressed a similar viewpoint, indicating that messages need to be more personal for youth to pay attention to them. One caregiver explicitly stated:

If you personalize it through an automated program [rather than a real person], it might feel kind of fake.

### End-User Testing Phase

On the basis of Development phase findings, the bidirectional SMS text-messaging intervention comprised 2 major components: (1) mental health or substance use treatment appointment reminders and (2) short motivational messages to enhance engagement and retention (Textboxes 1 and 2). Messages were intended to be simple and positive, and the theme of privacy, for example, was addressed by not including any language in texts, such as *mental health* or *substance use*.

A total of an additional 8 youth and 2 caregivers participated in the 6-month user-testing phase; 5-month survey data were available for 7 youth and 2 caregivers (1 youth only completed the 1-month survey). All youth received SMS text-messaging services for an average of 180.25 days (approximately 6 months). Of the 7 youth who completed a 5-month survey, all reported that the SMS text-messaging intervention helped them attend their treatment appointments. The feature that youth liked the most was being able to read messages at their convenience (n=6), followed by the ability to save reminders on their phone (n=4). The most helpful part of the SMS text-messaging intervention was that it reminded youth of appointments *they had completely forgotten about* (n=5). Most youth liked the motivational SMS text messages (n=5), felt they were supportive (n=5), and felt they were relevant (n=5). The majority of youth (n=6) felt that the motivational messages made them want to attend their counseling appointments. Youth did not feel any changes to the SMS text-messaging intervention were needed, and all would recommend it to a friend who is in counseling.

Both caregivers found the messaging system easy to use. Like youth, caregivers appreciated being able to read messages at their convenience and felt that the messages helped them remind their child of their upcoming appointments. They also liked the wording or tone of the SMS text messages, and they liked the message frequency. They agreed that the motivational messages made them want to encourage or support their child in attending their appointments. Neither caregiver felt that any aspect of the SMS text-messaging intervention should be changed.

**Sample motivational messages sent to youth.**

- Taking care of yourself includes making it to your appointments. Be sure to take care of YOU!
- Staying on top of your appointments is a great first step to keeping healthy habits
- Keep your head up—stay positive
- Always remember, you are braver than you believe, stronger than you seem
- Each appt. you attend will bring you closer to reaching your goals
- Your success in the future=making it to your appts. in the present

**Sample motivational messages sent to caregivers.**

- Remember to take care of you in the midst of taking care of your child
- Encouraging your child to stay on top of their appointments will help them keep healthy habits
- One small positive thought can change your whole day
- The struggle you are in today is developing the strength you need for tomorrow. Keep moving forward!
- Helping your child get to their appts. Will bring them closer to their goals
- Your child’s future success=making it to their appts. in the present

## Discussion

### Principal Findings

Our small first-time pilot exploration led to 2 overarching findings. First, the bidirectional use of informal SMS text messaging between JJ personnel and youth (and caregivers) in their caseloads is acceptable and commonly occurring; however, current SMS text-messaging practices are nonsystematic and idiosyncratic, depending on a multitude of individual, family, or system-level factors. Second, a formal SMS text-messaging service provided for 6 months to youth on probation, which comprises behavioral health appointment text reminders and motivational SMS text messages, appears feasible to implement and acceptable to end users.

JJ personnel, caregivers, and youth shared information that not only informed the development and content of our pilot SMS text-messaging service but also provided ideas for future research. For example, JJ personnel and caregiver stakeholders shared that utilizing SMS text messaging for quick *check-ins* as well as brief reminders for upcoming important appointments (including treatment and counseling, court, school, and job) was frequent and generally accepted by all stakeholders (including youth) as helpful, supportive, and much easier than utilizing phone or in-person strategies for reminders and engagement. Youth were also clear that messages should be brief and not contain any stigmatizing language or reveal sensitive information. Some youth verbalized a preference for visual (eg, MMS) messaging content as part of reminders and motivational check-ins. Youth and JJ personnel also wanted the messaging to be tailored and bidirectional and not rote or automatic. Incorporating some of these elements into the piloted SMS text-messaging service may have been responsible for youth and caregiver’s high acceptability and satisfaction with the SMS text-messaging program. The challenge for future research and implementation may be to identify how to create chatbots or other highly tailored ways of engaging youth that do not require 1:1 24/7 human resources (as our service did) but still provide the experience of tailoring and personalization. Similarly, identifying whether MMS content is more engaging and effective than SMS content and whether there is certain content that is more engaging for caregivers than for youth are all important areas for future research.

Perhaps of most notable significance to informing future digital health research and practice in this area was JJ personnel’s recognition of the multiple systems-level factors they confront when informally texting with youth. Without clear policy, guidance, and protocol on acceptable SMS text-messaging practices with justice-involved youth and within justice settings, such as probation, JJ personnel are unsure about critical issues related to privacy, confidentiality, and stigma. Examples include the following: (1) what aspects of current information-sharing (eg, through email, phone, and release of records) practice apply to texting, (2) what constitutes self-incriminating information via text (eg, if youth inadvertently admit to using a substance and communicating it via an SMS text message to their probation officer), (3) what and when can texts be subpoenaed, (4) how can one confirm that the private information being shared via text is arriving to the intended recipient, (5) how to or whether to document texting and information contained within texts in legal or clinical care records (as collateral), and (6) how to exert boundaries around texting (eg, what responsibility does a probation officer have to respond back to youth texting late at night about being in an emergent, unsafe, or illegal situation?). This pilot study utilized an external SMS text-messaging platform with aspects such as timing, frequency, and content standardized across participants. Whether the decision is made by JJ systems and partnering providers to utilize external SMS text-messaging service supports or to develop internal policies and procedures regarding the use of informal SMS text messaging with youth and families, these complex issues must be addressed. Future research that explores these ethical questions and other system- and policy-level factors associated with barriers to and facilitators of using a digital health intervention to improve justice-involved youth’s behavioral health outcomes is a key next step in the field.

### Limitations and Future Directions

This first, small pilot study starts an important scientific conversation about use of digital health technology to improve mental health and substance use treatment engagement (and subsequently outcomes) for this vulnerable youth population, but the study is not without its limitations. Data collection was limited to a small number of stakeholders and end users in 1 small geographical region of the United States; however, incorporation of perspectives from youth, caregivers, *and* JJ personnel allowed us to triangulate across key stakeholders and helped us begin to identify commonalities and differences in various stakeholders’ perception of what would be acceptable and feasible. JJ personnel included frontline probation and provider staff but not higher-level administration and decision makers with respect to policy- and system-level changes; given our preliminary findings, future research will want to consider how to add this level of stakeholder perspective, given they would be key to implementation. Overall, our data suggest that it is not clear that, in day-to-day practice, all youth are getting the texting and reminders and support, and our preliminary findings suggest that text messaging is not done in any sort of systematic or consistent way, which will ultimately, differentially impact outcomes. The user testing data provide some initial demonstration that it is feasible and acceptable for youth and caregivers to receive these SMS text messages (as part of an outside service) to remind and motivate them to attend treatment appointments, but what remains unknown is whether such an intervention can lead to improved youth outcomes. Following youth to measure (in more detail) current texting practices and the preliminary impact of messaging on youth treatment engagement was not a part of this pilot study. Ultimately, the impact of SMS text messaging on rates of appointment compliance, treatment attendance, and school absenteeism remains an empirical question. Do these SMS text-messaging reminders actually lead to improved adherence to scheduled appointments, such as with a case manager, a court hearing, or mandated substance use counseling? Is that outcome moderated by who sends the SMS text message (ie, probation officer vs treatment provider) or is there an additive, enhanced effect of getting coordinated reminders from all those involved in the youth’s care (eg, to demonstrate communication across systems and strong care coordination)? When the caregiver is also included in these SMS text messages, does this increase the likelihood of more positive youth attendance and engagement outcomes? Examples of future directions therefore include the following: (1) an efficacy trial of this SMS text-messaging intervention with appointment reminders and motivational messages to enhance treatment engagement to examine actual impact on outcomes (eg, does the SMS text messaging actually lead to increased treatment attendance, fewer symptoms, and lower rates of recidivism), (2) identifying the systems and organizational factors associated with uptake of such intervention, and (3) study of the ethical issues associated with the potential for self-incrimination, information-sharing, and other privacy concerns associated with sharing information with youth and caregivers over texting. These are all important next steps for this nascent area of highly significant digital mental health research.

## Abbreviations

CINI: court-involved, nonincarcerated
JJ: juvenile justice
mHealth: mobile health
MMS: Multimedia Messaging Service
NIH: National Institutes of Health
PI: principal investigator
SMS: short message service
